# Polyethylenimine as a Non-Innocent Ligand for Hexacyanoferrates Immobilization

**DOI:** 10.3390/molecules27238489

**Published:** 2022-12-02

**Authors:** Denis Balatskiy, Ivan Tkachenko, Irina Malakhova, Natalia Polyakova, Svetlana Bratskaya

**Affiliations:** Institute of Chemistry, Far Eastern Branch of Russian Academy of Sciences, 159 Prosp00-Letiya Vladivostoka, 690022 Vladivostok, Russia

**Keywords:** hexacyanoferrates, ferrocyanides, cyano-bridged complexes, coordination polymers, hybrid materials, polynuclear complexes, polyethylenimine, polyamine ligands, molecular magnets

## Abstract

To understand how polyethyleneimine (PEI), as a ligand, affects structure and properties of the transition metals hexacyanoferrates (HCFs) immobilized in cross-linked PEI matrix, we have synthesized Cu(II), Zn(II), and Fe(III) HCFs via successive ion-exchange reactions with metal salts and K_4_[Fe^II^(CN)_6_] or K_3_[Fe^III^(CN)_6_]. The structure and properties of the obtained materials in comparison with the crystalline HCF analogs were investigated with FT-IR, Mössbauer, and UV–Vis spectroscopy. Complete reduction of Fe(III) to Fe(II) by PEI in HCF(III) was confirmed. When synthesis was performed at pH favoring binding of precursor metal ions by PEI, cyano-bridged hybrids rather than polymer—HCFs composites were formed. Although the obtained hybrids did not demonstrate sorption activity toward cesium ions, known for crystalline HCFs, they are of interest for the other applications. SQUID measurements revealed a significant difference in magnetic properties of PEI–HCFs hybrids in comparison with crystalline HCFs. Due to the Fe(III) to Fe(II) reduction in HCF ions, Cu(II) and Fe(III) HCFs(III) lost the molecular magnets properties in PEI matrix, but magnetic ordering, including ferromagnet-antiferromagnet interactions, was observed in all hybrids over the broad temperature range.

## 1. Introduction

Polymers are widely used in fabrication of versatile hybrid materials to control growth, prevent aggregation, and drive assembly of metal [1,2,3], metal oxides [4,5], and other types [6,7,8,9] of nanoparticles. Interactions between nanoparticles and polymers, especially those with chelating and redox active moieties, can significantly affect functional properties of their hybrids. Zn–N interactions in polyethylenimine (PEI)-containing ZnO/PbS heterojunctions were suggested to be responsible for the better crystallization, reduced work function of ZnO and increased the built-in voltage that improved carrier separation in solar cells [4]. Modification of carbon nanotubes with PEI allowed decreasing the size of Pd nanoparticles, changing the electronic state of Pd, and enhancing the hydrophilicity of the catalyst for the dehydrogenation of formic acid [2]. Highly stable dendrimer-protected silver nanoclusters with narrow size distribution were obtained using a third-generation poly(propyleneimine) dendrimer as both a reducing agent and a protective agent [10]. Production of high-resolution microstructures on silicon, glass, or polystyrene was achieved by combination of electrostatic deposition on PEI-coated substrates followed by silver crystallization on the deposited silver nanoparticle agglomerates [1].

Hybrids based on Prussian Blue (PB) analogs serve as a striking example of the organic-inorganic materials, whose properties can be controlled by selection of the polymer. Additional ligands in PB analogs and related cyano-bridged polymers can mediate interactions between metal ions in M′-CN-M pairs [11] and tune materials characteristics for application as magnetic/photomagnetic/energy storage devices, sensors, catalysts, sorbents, nanozymes et cet. [12,13,14]. Depending on the field of application, efforts were made to obtain soluble PB analogs [15,16] or cyano-bridged nanoparticles homogeneously dispersed in polymer matrix [17,18]. Uemura et al. were the first who reported the effect of the surface environments on the charge transfer (CT) from Fe(II) to Fe(III) in PB nanoparticles capped with several polymers [16]. Earlier the size-dependent magnetic properties of PB nanoparticles stabilized by poly(vinylpyrrolidone) were demonstrated by the same group [15].

Reduction of [Fe(CN)_6_]^3−^ to [Fe(CN)_6_]^4−^ by PEI [6] or chitosan hydrolysis products [7] was observed for PB nanoparticles synthesized in acidic polymer solution using K_3_[Fe(CN)_6_] as a single precursor. PEI-mediated synthesis of mixed Cu–Fe and Ni–Fe hexacyanoferrate nanoparticles with good peroxidase mimetic activity was also performed in acidic media using K_3_[Fe(CN)_6_] as an iron source [19]. At the same time, cyano-bridged networks formed in pores of chitosan by consecutive treatment of the polymer matrix with metal salts and (N(C_4_H_9_)_4_)_3_[Fe^III^(CN)_6_] solutions in methanol yielded magnetic composites with color of the corresponding bulk hexacyanoferrate [17] that confirmed preservation of the Fe(III) oxidation state through the synthesis. Polymer–PB-analog composites obtained by the similar route in an alginate matrix using acetonitrile as a solvent demonstrated superparamagnetic, spin-glass or paramagnetic behavior typical for the corresponding PB analog nanoparticles [18].

Control of iron oxidation state in transition metals hexacyanoferrates is also important for the efficient cesium sorption [20]. When [Fe(CN)_6_]^4−^ was converted to [Fe(CN)_6_]^3−^ in nitric acid solution, the efficiency of cesium uptake with Chelex-20 resin containing copper hexacyanoferrate dropped notably, but was recovered in the presence of reducing agents [21]. In most cases, hexacyanoferrates immobilization in organic ion-exchangers or encapsulation/co-precipitation using natural and synthetic polymers yields composite materials with properties similar to those of the crystalline hexacyanoferrates [14,22]. The lower sorption capacity of composites due to the dilution of reactive sites in the matrix and slower sorption kinetics due to diffusion limitations are compensated by the significantly improved efficiency of sorbents separation [14]. Despite the large number of successful examples of polymer-hexacyanoferrates composite sorbents, we have surprisingly failed to fabricate composite sorbent using PEI cryogels as a matrix for Cu(II) hexacyanoferrate immobilization [23]. Later, we have shown that Zn(II) nanoparticles formed immediately after addition of K_4_[Fe(CN)_6_] to the solution of PEI−Zn(II) complex were dissolved within a few minutes, so that the fabrication method had to be carefully adapted to obtain an efficient composite for cesium uptake [24].

We have assumed that this remarkably different behavior of PEI in polymer mediated synthesis of hexacyanoferrates can be related to formation of cyano-bridged polymers (hybrid materials) with new properties, which do not possess sorption characteristics of crystalline hexacyanoferrates [23]. This assumption was not proved, but understanding how PEI, as a ligand, affects structure and properties of the immobilized hexacyanoferrates is important for the targeted development of functional materials for various fields of application. Here we have investigated in detail the PEI role in fabrication of insoluble hybrid materials containing hexacyanoferrates and related soluble cyano-bridged polymers with several transition metal ions and [Fe(CN)_6_]^3−^ or [Fe(CN)_6_]^4−^ as precursors.

## 2. Results and Discussion

### 2.1. Polyethyleneimine-Hexacyanoferrate (PEI–HCF) Hybrids with Cross-Linked Polymer Matrix

#### 2.1.1. Fabrication, Composition and Sorption Properties

To elucidate reasons leading to the drastic decrease of cesium sorption activity of hexacyanoferrates (II) (HCF(II)) after immobilization in PEI matrix [23], we have synthesized a series of materials using PEI cross-linked with 1,4-butanediol diglycidyl ether [25] as a support. In contrast to the earlier reported by our group monolithic composite materials [24], the inorganic phase was immobilized to the fine fraction of cross-linked PEI via successive contact steps with metal salts and K_3_[Fe(CN)_6_] or K_4_[Fe(CN)_6_] in solution under static conditions. This route is typical for the HCFs immobilization in polymer matrices or ion-exchange resins [14], when the content of inorganic phase in the composite is determined by the ion-exchange capacity of the matrix. The sorption capacity of PEI decreases in the order Cu(II) > Zn(II) > Ni(II) > Co(II) [26], so that copper and zinc salts were used as precursors providing potentially highest content of HCFs in the material. Another reason to choose these metal ions consists in significantly higher binding constant for Cu(II) over Zn(II) ions in PEI solution [27] that can be used to verify our hypothesis that formation of highly stable PEI complexes with metal ions prevents formation of crystalline HCFs.

Table 1 demonstrates that all PEI–HCFs hybrids showed low efficiency of cesium uptake in comparison with the crystalline HCF analogs. The maximal sorption activity was observed for Zn(II) HCFs, regardless of the anion precursor type—(K_3_[Fe(CN)_6_] or K_4_[Fe(CN)_6_]). Analysis of the elemental composition of the crystalline HCFs and related PEI–HCF hybrids (Table 1) reveals the following features: (i) potassium was either not detected or present in PEI–HCF hybrids in very low content, although it was found in the PEI–Zn(II)HCF(II) monolith composite obtained earlier using another fabrication method [24]; (ii) Zn:Fe atomic ratios were close to 2:1 for both PEI–Zn(II)HCF(II) and PEI–Zn(II)HCF(III) hybrids suggesting that Fe(III) to Fe(II) reduction occurred during synthesis and PEI–Zn(II)HCF(II) hybrids were obtained using both anionic precursors; (iii) Cu:Fe atomic ratios in PEI–Cu(II)HCFs were higher than was expected for both HCF(II) and HCF(III), despite the excess of anionic precursors was added during synthesis. Furthermore, PEI–Cu(II)HCFs had, untypical for the crystalline analogs, dark blue color. To elucidate the structure of PEI–HCF hybrids in detail, materials were investigated using the FT-IR and Mössbauer spectroscopy.

#### 2.1.2. Mössbauer Spectroscopy

**Figure 1:**
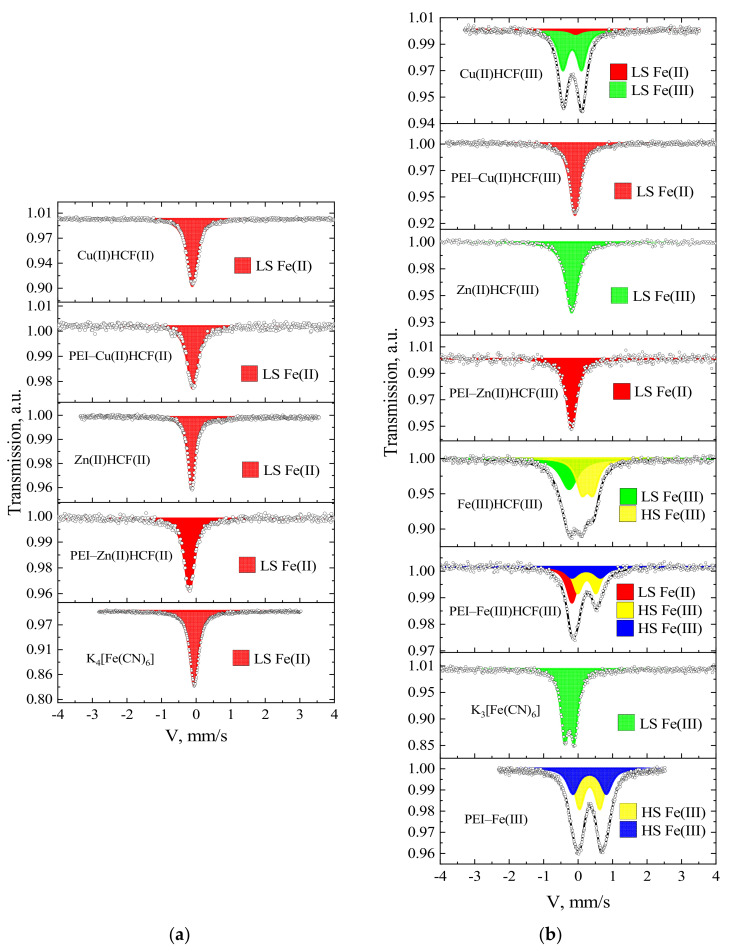
Mössbauer spectra of crystalline hexacyanoferrates (HCF) and related PEI–HCF hybrids obtained using K_4_[Fe^II^(CN)_6_] (**a**) and K_3_[Fe^III^(CN)_6_] (**b**) as precursors: dots are experimental data; lines are spectra fits obtained with the WinNormos program.

Although isomer shifts between HCF(II) and HCF(III) have only a small difference of about 0.05 mm/s in Mössbauer spectra, it is sufficient to differentiate them [28]. Spectra of the crystalline Cu(II) and Zn(II) HCFs demonstrated typical for HCF(II/III) features (Figure 1, Table 2).

In agreement with the literature data [29,30], singlets with isomer shifts of −0.10 and −0.12 mm/s were found for Cu(II) and Zn(II) HCFs(II), respectively. The spectrum of Cu(II)HCF(III) is represented by an asymmetric doublet, which, according to [31,32], was fitted with two components: a singlet with isomer shift of −0.07 mm/s, corresponding to low spin Fe(II) in cyano-bridge Fe^II^–CN–Cu^II^, and a doublet with isomeric shift −0.16 mm/s and quadrupole splitting of 0.55 mm/s, corresponding to low spin Fe(III) in Fe^III^–CN–Cu^II^. A singlet in Zn(II)HCF(III) spectrum with an isomeric shift of −0.17 mm/s corresponds to low spin Fe(III) in the Fe^III^–CN–Zn^II^ bridge [33,34]. The Fe(III)HCF(III) spectrum was fitted with two components: a singlet with an isomeric shift of −0.15 mm/s corresponding to low spin Fe(III) in HCF (III) ion (Fe^III^–CN) and a doublet with parameters δ = −0.39 mm/s and Δ = 0.34 mm/s, which can be assigned to the high spin Fe(III) in outer-sphere (Fe^III^–NC).

Analysis of Mössbauer spectra of the PEI–HCF hybrids (Table 2) unambiguously confirmed that (i) hexacyanoferrate anions were not decomposed during synthesis; (ii) only one position with isomer shift of 0.08 ± 0.01 mm/s and linewidth of 0.36 ± 0.01 mm/s, corresponding to the low spin state of Fe(II) in hexacyanoferrates (II) [28,29], was detected that indicates complete reduction of [Fe^III^(CN)_6_]^3−^ to [Fe^II^(CN)_6_]^4−^ in PEI–HCF hybrids. Thus, the Mössbauer spectra of hybrids, which were supposed to contain Zn(II) and Cu(II) HCF(III), were identical to those of crystalline Zn(II) and Cu(II) HCFs(II) and corresponding PEI–HCF(II) hybrids (Figure 1, Table 2).

The Mössbauer spectrum of PEI–Fe(III)HCF(III) hybrids could not be fitted with two components corresponding to the doublet (δ = 0.36 and Δ = 0.53 mm/s) of high spin Fe(III) in the outer-sphere and the singlet (δ = −0.07 mm/s) of low spin Fe(II) in hexacyanoferrate ion (Table 2). The third necessary component—a doublet with δ = 0.35 and Δ = 0.83 mm/s can be assigned to the high-spin Fe(III) in hydroxy complexes [35]. Analysis of PEI–Fe(III) complex, which was obtained after Fe(III) sorption on PEI at the first stage of hybrid fabrication, revealed the presence of the same components of high-spin Fe(III). This is not surprising, since Fe(III) hydrolysis starts near pH 3. Nevertheless, in the used route of the hybrids’ fabrication, pH was not lowered to pH 2 to avoid hydrolysis, since we have intentionally chosen conditions, at which PEI binds metal ions (pH > 4).

#### 2.1.3. FT-IR Spectroscopy

**Figure 2:**
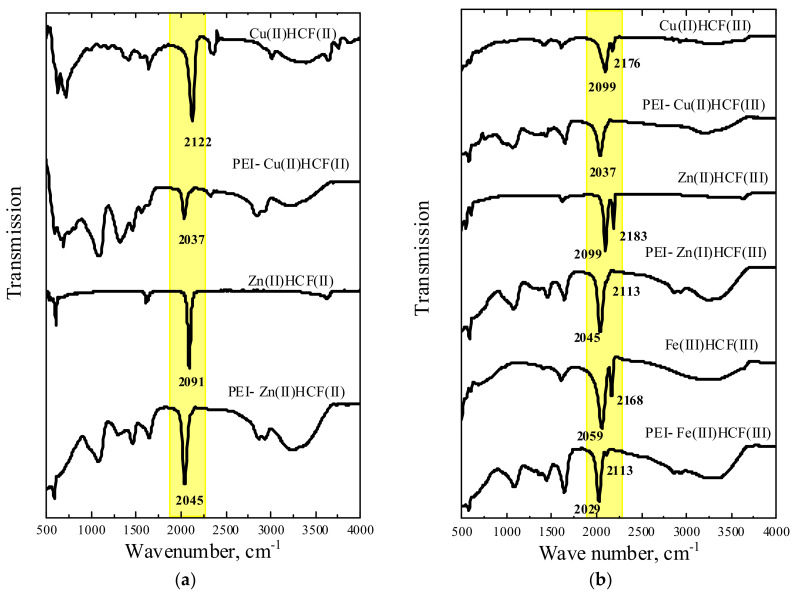
FT-IR spectra of crystalline hexacyanoferrates (HCF) and related PEI–HCF hybrids obtained using K_4_[Fe^II^(CN)_6_] (**a**) and K_3_[Fe^III^(CN)_6_] (**b**) as precursors.

Figure 2 shows a good correlation between the obtained and reported-in-literature FT-IR spectra of crystalline HCFs (II) and (III) [28,31,34,36,37]. Two ν(CN) bands at 2099 and 2176 cm^−1^ in Cu(II)HCF(III) spectrum correspond to Fe^II^–CN–Cu^II^ and Fe^III^–CN–Cu^II^, respectively [31,34]. Assignment of ν(CN) bands at 2187 cm^−1^ to Fe^III^–CN–Zn^II^, and at 2099 cm^−1^ to Fe^II^–CN–Zn^II^ was performed as in [28], two bands with the close frequencies were also reported for Zn(II)HCF(III) in [33,37,38]. However, Fe^II^–CN–Zn^III^ motives were not detected by Mössbauer spectroscopy (Table 2). FT-IR spectrum of Fe(III)HCF(III) with ν(CN) bands at 2059 and 2168 cm^−1^ can be assigned to the formation of Berlin’s green, for which ν(CN) at 2172 and 2083 cm^−1^ were reported [36]. In [37], the bands at 2160 cm^−1^ and 2071 cm^−1^ were assigned to Fe(III) HCF(III) and HCF(II), respectively. It should be mentioned that different forms of Fe(III) hexacyanoferrates with mixed valence states are known [38], so that a mixture of the products could be obtained.

FT-IR spectra of PEI–HCF hybrids confirmed the conclusion made using Mössbauer spectroscopy data on complete reduction of [Fe^III^(CN)_6_]^3−^ to [Fe^II^(CN)_6_]^4−^ in all materials. FT-IR spectra of Zn(II) and Cu(II) PEI–HCF(III) (Figure 2b) were identical to those of PEI–HCF(II) (Figure 2a) hybrids but, in contrast to Mössbauer spectra, a significant difference was observed between hybrids and crystalline HCFs (Figure 2). In is known that particularly strong bonding of the Cu(II) to the N end of the CN group results in the highest *ν*(CN) vibration frequency in Cu(II) hexacyanoferrates of divalent metals [39]. However, receiving electrons from PEI favors copper electronic configuration close to 3*d*^10^ and weakens Cu(II) binding to the CN ligand in the PEI–Cu(II)HCF(II) hybrid. This results in ν(CN) shift to the lower vibration frequencies for 85 cm^−1^. This effect was also observed for PEI–Zn(II)HCF(II) hybrid. However, due to the weaker Zn(II) binding to PEI [27], ν(CN) band shift was less pronounced (46 cm^−1^). The band at 2029 cm^−1^ in the FT-IR spectrum of PEI–Fe(III)HCF(III) hybrid can be assigned to ν(CN) band reported for PB at 2084 cm^−1^ in [6] and 2086 cm^−1^ in [16], assuming shift to the lower frequency due to coordination of Fe(III) with PEI. It should be emphasized that in the inner sphere of HCF only low spin Fe(II) was found by Mössbauer spectroscopy for this hybrid. A low intensity band at 2113 cm^−1^ can be assigned to the outer-sphere Fe(III). A shoulder at 2113 cm^−1^ was also observed in PEI–Zn(II)HCF(II) FT-IR spectrum suggesting the possible presence of Fe(III) in the outer sphere, although it was not unambiguously detected by Mössbauer spectroscopy.

#### 2.1.4. Magnetic Properties

**Figure 3:**
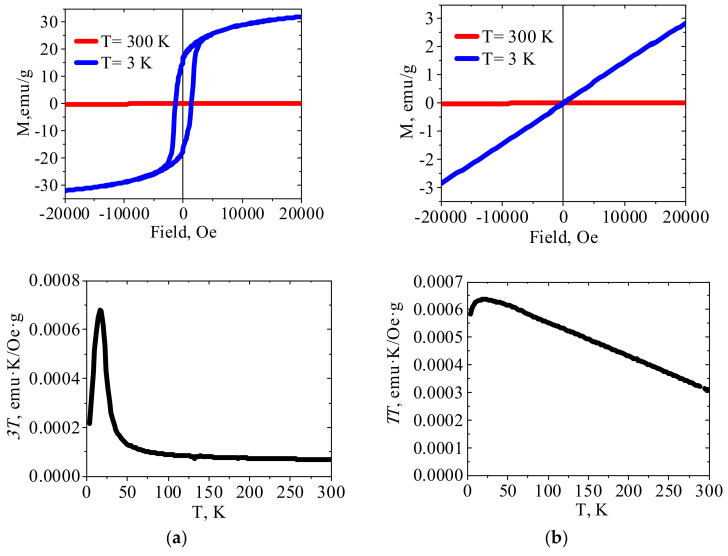
The magnetization curves and *χT* temperature dependences for (**a**) crystalline Cu(II)HCF(III) and (**b**) PEI–Cu(II)HCF(III) hybrid (please refer to Figure S3, Supplementary Materials for field dependence of PEI–Cu(II)HCF(III) hybrid magnetization at room temperature).

**Figure 4:**
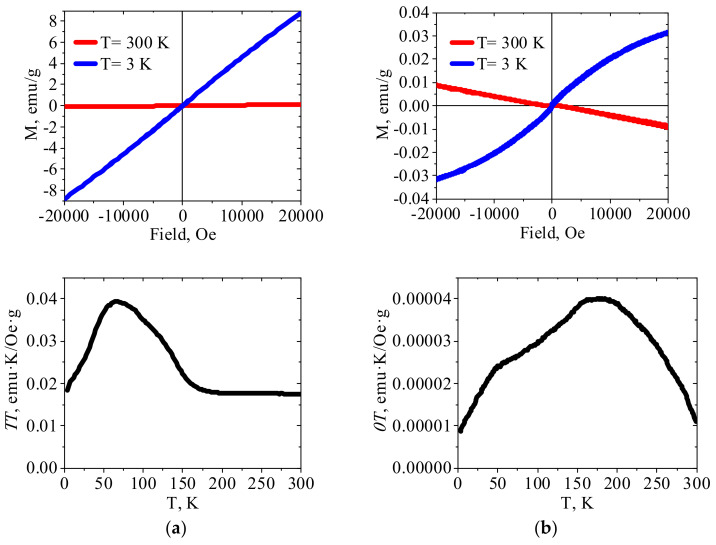
The magnetization curves and *χT* temperature dependences for (**a**) crystalline Zn(II)HCF(III) and (**b**) PEI–Zn(II)HCF(III) hybrid.

**Figure 5:**
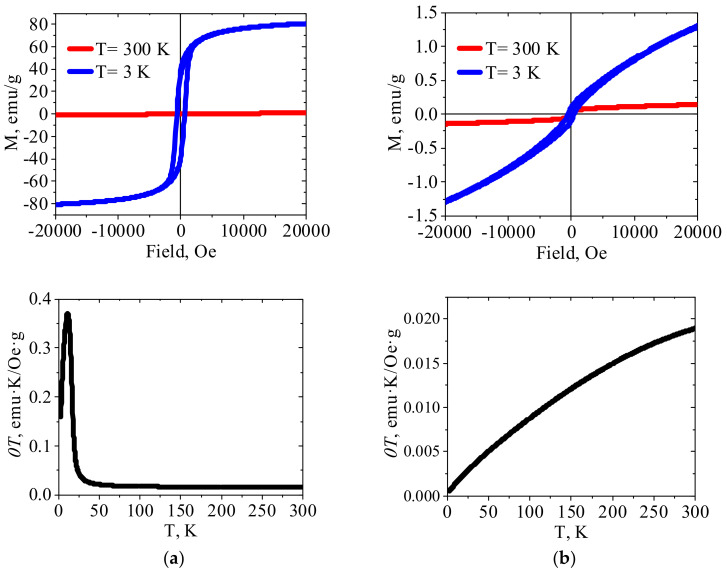
The magnetization curves and *χT* temperature dependences for (**a**) crystalline Fe(III)HCF(III) and (**b**) PEI–Fe(III)HCF(III) hybrid.

Figure 3, Figure 4 and Figure 5 show the results of SQUID measurements for crystalline HCFs(III) and related PEI–HCF hybrids at various external fields and temperatures. XRD data for these compounds are presented in Figure S1 (Supplementary Materials). Temperature dependences of magnetization are shown in Figure S2 (Supplementary Materials). In agreement with the literature data, Cu(II)HCF(III) and Fe(III)HCF(III) exhibited properties of the molecular magnets with Curie temperatures of 19 K [32] and 22 K [40], respectively. The Curie temperature of 22 K was reported for Prussian green [40], so that the assumption on the Fe(III)HCF(III) structure from FT-IR spectrum is supported by the magnetic properties. The Curie temperature of 5.6 K was reported for Prussian blue in [41]. It can be mentioned that long-range ferromagnetic ordering in PB (Fe^III^_4_[Fe^II^(CN)_6_]_3_·xH_2_O) was reported by Mayoh and Day [42], who explained it by ferromagnetic superexchange between high spin Fe(III) mediated via CN bridge involving diamagnetic low spin Fe(II) centers.

According to the literature data [32], crystalline Zn(II)HCF(III) is paramagnetic, so linear dependence of *χT* vs. T was expected. However, Figure 4a shows that *χT* value was virtually constant in the temperature range 300–180 K, increased smoothly during cooling down to approximately 66 K, and then gradually decreased. This type of *χT* vs. T curve indicates transition to another magnetically ordered state in Zn(II)HCF(III) at a temperature of 66 K and, according to [43], these interactions are antiferromagnetic. The relatively strong magnetic interactions between distant iron(III) atoms in zinc hexacyanoferrates were possible due to the charge delocalization through the CN bridges [43]. It should be emphasized that crystalline HCFs were intentionally precipitated at pH value close to 5, at which PEI–HCF hybrids were fabricated. Thus, the reported magnetic properties must be considered taking into account synthesis conditions, FT-IR and Mössbauer spectra, elemental composition and XRD patterns of the reported materials. One cannot also completely exclude minor impurities, e.g., iron (III) hydroxides with reported Néel temperature of 48 K [44] or intercalation of Fe(III) ions in Zn(II)HCF(III) lattice.

Comparison of magnetic properties of crystalline HCFs (Figure 3, Figure 4 and Figure 5a) and related PEI–HCF hybrids obtained via stepwise ion-exchange in cross-linked PEI matrix (Figure 3, Figure 4 and Figure 5a) shows that, in contrast to polymer-PB analog composites obtained in alginate [18] and chitosan [17] matrices, PEI induced magnetic ordering and affected temperature dependences of magnetic characteristics in all hybrids.

First of all, PEI reduced Fe(III) to Fe(II) in hexacyanoferrates (III), as was confirmed with Mössbauer spectroscopy (Figure 1, Table 2). Thus, immobilization of the molecular magnet Cu(II)HCF(III), which is reduced to Cu(II)HCF(II) in PEI matrix, expectedly resulted in a loss of the magnetic properties determined in Cu(II) HCF(III) by ordered 3d ferromagnetic network of co-existing cyano-bridged paramagnetic structures Fe^II^-CN-Cu^III^ and Fe^III^-CN-Cu^II^ [31]. Figure 3b shows for PEI–Cu(II)HCF(III) hybrid the increase of *χT* with the decreasing temperature down to 16 K, and an insignificant deviation from a straight line of the low-temperature field dependence. These data allow the conclusion that ferromagnetic interactions are realized in this hybrid over the entire temperature range. The possibility of such interactions in Cu(II) hexacyanoferrates(II) is confirmed by the data on heterometallic ion-pair complex containing ethylenediamine copper(II) complex cation and hexacyanoferrate anion, which showed the same type of *χT* vs. T dependence [45].

For the PEI–Fe(III)HCF(III) hybrid, in which Mössbauer spectroscopy shows the presence of only low spin Fe(II) in the inner sphere of hexacyanoferrate, *χT* value decreases upon cooling over the entire temperature range (Figure 5b). This behavior of *χT* vs. T plot indicates antiferromagnetic interactions in this material. The features of the field dependence of the magnetization at a temperature of 3 K are a sharp increase in the coercive force compared to room temperature, the absence of saturation even in the high-fields region, and the shift of the hysteresis loop to the region of negative fields. This type of the magnetization dependence on the external field is typical for materials with ferromagnet-antiferromagnet interactions. In other words, one can assume that two types of magnetic ordering are realized in PEI–Fe(III)HCF(III) hybrid. We can suggest formation in this hybrid multidimensional network structure, in which ferromagnetic interaction operates within each layer and the antiferromagnetic interaction operates between the layers, as was reported for Mn(III) Schiff base complexes with hexacyanoferrate(III) [46]. It shall be also mentioned that presence of Fe(III) hydroxides in this hybrid was detected with Mössbauer spectroscopy (Table 2).

*χT* vs. *T* plot for PEI–Zn(II)HCF(III) hybrid does not have a linear region as for the crystalline Zn(II)HCF(III), besides, maximum is shifted to the higher temperature (177 K). At the same time, a shoulder at a temperature of 48 K can be clearly identified. The absence of saturation on the M(H) curve obtained at 3 K, as well as a slight shift of the loop to the negative fields region, indicates ferromagnet/antiferromagnet interactions in this material, but weaker than in PEI–Fe(III)HCF(III) hybrid. It should be mentioned that Mössbauer spectroscopy did not show the presence of Fe(III) in the PEI–Zn(II)HCF(III) hybrid. However, the low-intensity bands at 2113 cm^−1^ in FT-IR spectra of PEI–Zn(II)HCF(III) and PEI–Fe(III)HCF(III) hybrids (Figure 3b) suggest that one cannot totally exclude presence of Fe(III) traces in the HCF outer sphere in this hybrid.

### 2.2. Cyano-Bridged PEI–HCF Complexes in Solution

Binding of Cu(II), Zn(II), and Fe(III) ions to PEI at pH 7 followed by addition of [Fe(CN)_6_]^3−^ or [Fe(CN)_6_]^4−^ was investigated in solution to compare optical properties of PEI–HCF hybrids with those of the HCFs colloids, obtained by precursors mixing at stoichiometric ratios in HCF(II) and HCF(III). In contrast to the earlier reported works on PEI–mediated fabrication of BP analogs, which were performed in highly acidic media at pH < 1 [6,19] and resulted in formation of crystalline nanoparticles protected with the polymer shell, we have synthesized PEI–HCF hybrids at pH, when stable metal-polymer complexes were formed with all cationic precursors.

**Figure 6:**
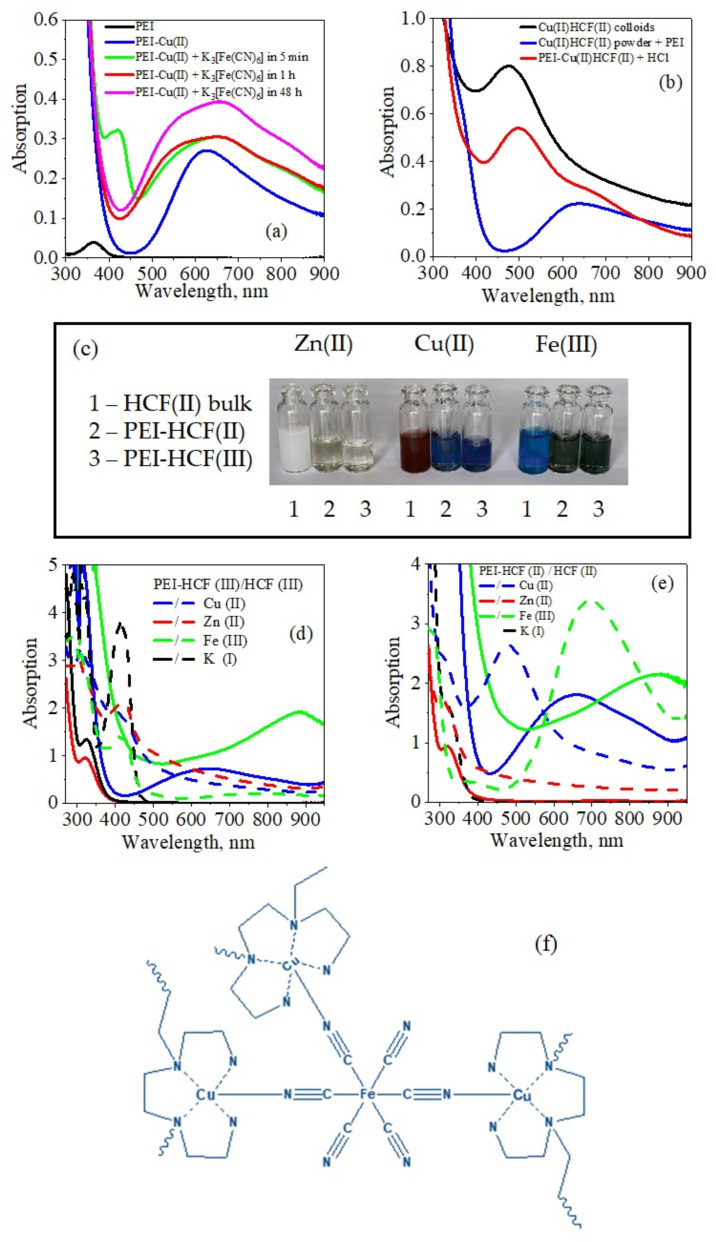
Optical properties of colloidal hexacyanoferrates (HCF) and related PEI–HCF hybrids: (**a**) evolution of electronic spectra of PEI–Cu(II)HCF(III) in solution; (**b**) reversible changes of optical properties after dissolution of crystalline powder of Cu(II)HCF(II) in PEI solution and after 4 M HCl addition to the solution of PEI–Cu(II)HCF(II) cyano-bridged complex; (**c**) photo of freshly precipitated HCFs(II) colloids and cyano-bridged complexes PEI–HCF(II) and (III) in solution; electronic spectra of colloidal hexacyanoferrates (HCF) and related cyano-bridged complexes obtained in solution using K_4_[Fe^II^(CN)_6_] (**d**) and K_3_[Fe^III^(CN)_6_] (**e**) as precursors. Hypothetical structure of PEI–Cu(II)HCF(II) cyano-bridged complex (**f**).

The reduction of [Fe(CN)_6_]^3−^ to [Fe(CN)_6_]^4−^, which was reported earlier in acidic PEI [6,19] and chitosan [7] solutions, was completed at pH 7 in PEI solution in less than 1 h. Thus, formation of PEI–HCF(II) was expected regardless of the oxidation state of iron in precursor anion in the same manner as was observed for the HCFs immobilized in cross-linked PEI matrix (Section 2.1). Figure 6 shows changes in the electronic spectrum of PEI–Cu(II) complex at L:M molar ratio of 10:1 after addition of [Fe(CN)_6_]^3−^ in the amount corresponding to Cu(II):Fe(III) molar ratio of 1.5:1. In 5 min we observed asymmetric broadening of PEI–Cu(II) absorption band at 625 nm and low-intensity absorption band at 420 nm. The latter band, which was assigned to the residual [Fe(CN)_6_]^3−^ ions, disappeared after 1 h, while the band in the range 420–900 nm demonstrated time-dependent increase of absorption intensity. The PEI–HCFs solutions were stable for at least 5 months.

The electronic spectrum of PEI–Cu(II)HCF(II), which was formed using [Fe(CN)_6_]^4−^ as a precursor, showed the same features as the PEI–Cu(II)HCF(III) spectrum (Figure 6b,d,e). This indicates that reduction of [Fe(CN)_6_]^3−^ to [Fe(CN)_6_]^4−^ resulted in formation of PEI–Cu(II)HCF(II) in both cases. Figure 6c demonstrates the remarkable difference in colors of PEI–Cu(II)HCF (deep blue) and Cu(II)HCFs colloids (reddish brown and pale dirty green for HCF(II) and (III), respectively). The shift of the absorption band from 480 nm for Cu(II)HCF(II) colloid to 659 nm for PEI–Cu(II)HCF(II) (Figure 6e) cannot be related to the effect of protective polymer shell in BP analogs nanoparticles, since in this case shift of the absorption band was reported to be within 30 nm [6,15].

The broad absorption band in the range 420–900 nm can be considered as a sum of sub-spectra with maximum at 659 nm and two shoulders at ~830 and ~560 nm (Figure 6a). Broad band in the visible range (590–780 nm) with low-energy shoulder at λ > 800 nm is typical for Cu^2+^ in square pyramidal geometry [47]. Earlier we have investigated Cu^2+^ binding to PEI using ESR spectroscopy and DFT calculations and showed that PEI–Cu(II) complex has geometry of distorted square pyramid, in which Cu(II) is five-coordinated [48]. The spectroscopic features very similar to those of PEI–Cu(II)HCF(II)in solution were reported for the ferrocyanide ion incapsulated by Cu(II) complexes with tripodal tetradentate ligands, when formation of heptanuclear and pentanuclear heterometallic assemblies was confirmed [49]. Thus, untypical for HCFs electronic spectra and strong deviations from stoichiometry in PEI–Cu(II)HCF(II)/(III) with a significant excess of Cu over Fe atoms (Table 1) suggest formation of polynuclear cyano-bridged complexes, whose hypothetical structure is shown in Figure 6f.

In visible light region of electronic spectra of both PEI–Fe(III)HCF(II) and PEI–Fe(III)HCF(III) the same broad absorption band at 880 nm was observed. Solutions were stable for several months and had deep greyish green color, although blue color typical for Prussian blue could be expected from the Mössbauer spectra of PEI–Fe(III)HCF(III) (Figure 1, Table 2). In this case, we also assume formation of cyano-bridged complexes, that explains why Fe(III)HCF(III), immobilized in cross-linked PEI matrix, does not show sorption activity for cesium ions, as it is known for the crystalline analogs [50].

It is interesting to note that adding HCl to PEI–Cu(II)HCF(II) solution leads to immediate dissociation of the PEI-Cu(II) complex and release of PEI and formation of Cu(II)HCF(II) colloids that is evident from change of blue color to purple-brownish, and shift of the absorption band to 497 nm (Figure 6b). The reversed route to the stable soluble PEI–Cu(II)HCF cyano-bridged complex from insoluble crystalline Cu(II)HCF(II) was also demonstrated. After addition of 5% PEI solution (pH 7) to the fine powder of crystalline Cu(II)HCF(II), crystals were dissolved in 1 min and solution turned blue showing the spectral features of PEI–Cu(II)HCF(II) cyano-bridged complex (Figure 6b). Addition of chitosan and carboxymethylchitosan, which were earlier used as matrices for fabrication of composite polymer-hexacyanoferrate sorbents [8,51], had no effect on solubility of HCFs.

Dissolution of the crystalline HCFs in PEI solution was also observed for Zn(II)HCF(II) and Fe(III)HCF(III). Although weaker binding of Zn(II) to PEI allowed earlier fabrication of composites with crystalline Zn(II)HCF(II) phase [24], the much longer fabrication time used here, most likely, led to the partial dissolution of crystalline nanoparticles and, thus, to the lower sorption activity of the hybrid (Table 1). The difference in fabrication conditions also affected the elemental composition: when monolith PEI cryogel was modified with Zn(II)HCF(II) under dynamic conditions mixed zinc/potassium hexacyanoferrate was formed [24].

## 3. Materials and Methods

### 3.1. Materials

Branched polyethyleneimine (PEI) of an average molecular weight of 25 kDa was purchased from Alfa Aesar. 1,4-butanediol diglycidyl ether was purchased from Sigma-Aldrich (Darmstadt, Germany). All other reagents were of analytical grade.

### 3.2. Fabrication of the Hybrids

Cross-linked PEI was obtained as described in [24] and dispersed with ultrasound to the fine fraction with particle sizes about 200 µ to assure good accessibility of the surface for ion-exchange reactions. 200 mg of the dry cross-linked PEI was put in contact with 200 mL of the solution, containing 400 mg/g of the metal precursor (Cu(II), Zn(II), or Fe(III)). The continuous stirring of the mixture for 72 h was conducted using a Biosan PSU-20i orbital shaker (Riga, Latvia) The material, which is further referred to as PEI–metal complex, was separated and thoroughly washed with distilled water. Metal contents in PEI were determined by atomic absorption spectroscopy using an AA-7000 Atomic Absorption Flame Emission Spectrophotometer (Shimadzu, Kyoto, Japan). At the second stage, the PEI matrix saturated with metal ions (PEI–metal complex) was put in contact with K_4_[Fe(CN)_6_] or K_3_[Fe(CN)_6_] solution at solid:liquid ratio 1:250 for 72 h under continuous stirring. The required content of the anion precursor was calculated, taking into account an excess over the immobilized metal ions as summarized in Table 1. After thorough washing with distilled water PEI–HCF hybrids were dried in vacuum at 40 °C.

The crystalline HCFs were obtained by mixing under the constant stirring of 0.1 M solutions of cationic and anionic precursors at a stoichiometric ratio at pH 5.0 ± 0.2. Precipitates were separated, washed with distilled water and dried in vacuum at 40 °C.

Formation of the PEI–HCF hybrids in solution was performed as follows: 0.5 M solution of metal salt was added to 5% PEI solution preliminarily adjusted to pH 7. The metal:PEI molar ratio was fixed at 1:10, the PEI monomer unit molecular weight was taken as 127 g/mol. Then, after 2 h K_4_[Fe(CN)_6_] or K_3_[Fe(CN)_6_] solution was added at stoichiometric ratio.

### 3.3. Characterization of HCFs and PEI–HCF Hybrids

The elemental compositions of powdered HCFs and PEI–HCF hybrids were determined by energy dispersive X-ray fluorescence spectroscopy using a Shimadzu EDX-800-HS spectrometer equipped with X-ray tube with Rh-anode; the exposure time was 100 s. The elemental composition was calculated using the spectrometer software without taking into account the content of light elements (H, C, N, O).

The Mössbauer spectra were obtained at room temperature using a Wissel (Ortenberg, Germany) spectrometer in transmission geometry and a ^57^Co(Rh) source. The Mössbauer spectra were fitted using the WinNormos program in order to obtain the values of isomer shift (*δ*), quadrupole splitting (Δ), linewidth (*Γ*), and relative sub-spectrum area. The velocity scale was calibrated using the spectrum of metallic iron (α-Fe). The value of isomer shifts was determined relatively to the center of gravity of the α-Fe spectrum.

Fourier transform infrared (FT-IR) spectra were recorded using an IR Affinity-1 spectrometer with a MIRacle 10 FT-IR accessory (Shimadzu, Kyoto, Japan).

X-ray powder diffraction analysis (XRD) of HCFs and PEI–HCF hybrids was carried out using multipurpose diffractometers STOE STADI P (STOE & Cie GmbH, Darmstadt, Germany) and RIGAKU SmartLab 9 kW (Rigaky Corporation, Tokyo, Japan) with Bragg-Brentano geometry (Cuλ2-irradiation, Ni-filter), respectively.

The materials magnetization was measured using an MPMS 7XL SQUID magnetometer (Quantum Design, San Diego, California, USA) The dependencies of the magnetization on temperature were measured at a magnetic field strength of 10 kOe, the cooling rate was 1 K/min. The measurement range was 300–3 K, the measurement increment was 2 K. The magnetic field dependencies were obtained at temperatures of 300 and 3 K in the range ± 20 kOe. Measurements were performed with increment of 100 Oe up to 1 kOe and of 500 Oe above 1 kOe.

Electronic spectra of HCF colloids and PEI–HCF complexes in solution were recorded using a UV-2600PC scanning UV–Vis spectrophotometer (Shimadzu, Kyoto, Japan) in a quartz cuvette with optical length of 1 cm after 30-fold dilution with distilled water.

The efficiency of the Cs^+^ ion uptake by the HCFs and PEI–HCF hybrids was determined in batch as follows: 5 mg of the material was stirred for 72 h with 5 mL of CsCl solution containing 20 mgCs/l (pH~6) at 200 rpm using a Biosan PSU-20i orbital shaker (Riga, Latvia). The efficiency uptake was calculated using the difference in Cs initial and equilibrium concentrations determined by atomic absorption spectroscopy using an AA-7000 Atomic Absorption Flame Emission Spectrophotometer (Shimadzu, Kyoto, Japan).

## 4. Conclusions

To understand why Cu(II), Zn(II), and Fe(III) hexacyanoferrates (HCFs) immobilized in cross-linked polyethyleneimine (PEI) matrix showed unexpectedly low efficiency of cesium uptake, we have investigated composition, structure and magnetic properties of PEI–HCF hybrids and their polymer-free crystalline analogs using energy dispersive X-ray fluorescence, FT-IR and Mössbauer spectroscopy. UV–Vis spectroscopy was applied to investigate optical properties of PEI–HCF complexes in solution.

It was found that Fe(III) to Fe(II) reduction by PEI in hexacyanoferrates (III) resulted in the formation of PEI–HCF(II) hybrids, when both K_4_[Fe^II^(CN)_6_] and K_3_[Fe^III^(CN)_6_] were used as precursors. Strong deviation from stoichiometry with a significant excess of Cu over Fe atoms in PEI–Cu(II)HCF(II) hybrid and its unusual for crystalline Cu(II) hexacyanoferrates dark blue color in solution led us to the suggestion that strong binding of metal ions to PEI resulted in formation of cyano-bridged complexes rather than polymer-capped crystalline HCFs, which were earlier obtained in PEI–mediated PB synthesis at pH < 1. This suggestion was supported by the strong shift of the CN vibration band in PEI–HCFs in comparison with crystalline HCFs and electronic spectra, similar to those reported for polynuclear cyano-bridged Cu(II) complexes with tripodal tetradentate ligands. Furthermore, liberation of PEI from PEI–Cu(II)HCF(II) complex in highly acidic media resulted in formation of reddish-brown Cu(II)HCF(II) colloids, and, oppositely, crystalline Cu(II)HCF(II) were readily dissolved in PEI solution demonstrating dark blue color of cyano-bridged complex. Similar effects were observed for the hybrids with Zn(II) and Fe(III) HCFs.

Although the formation of cyano-bridged complexes instead crystalline HCFs dispersed in polymer matrix limits PEI application for fabrication of composite sorbents for cesium, the obtained hybrids demonstrate other interesting functional properties. SQUID measurements have shown that charge delocalization in CN bridge in PEI–HCFs hybrids induced magnetic interactions unusual for crystalline HCFs. The dependence of magnetization on the external field for PEI–Zn(II)HCF(II) and PEI–Fe(III)HCF(II) hybrids is typical for materials with ferromagnet-antiferromagnet interactions. Thus, PEI can be used as a ligand mediating formation of magnetic 2D coordination polymers and 3D assemblies of bimetallic networks with precisely controlled stoichiometry and spatial distribution of the components.

## Figures and Tables

**Table 1:** Elemental analysis and cesium sorption efficiency and ν(CN) of crystalline hexacyanoferrates (HCF) and related PEI–HCF hybrids, M—Cu(II), Zn(II), Fe(III).

Sample	Molar Ratios of Reagents M:Fe	Atomic Ratios in Product M:K:Fe	M ^1^, mg/g	Cu/Zn, at %	Fe, at %	K, at %	Efficiency of Cs^+^ Uptake ^2^, %
Cu(II)HCF(II)	2:1	1.79:0.42:1		55.76	31.15	13.08	99
PEI–Cu(II)HCF(II)	1:1	3.23:0.19:1	140	75.01	21.01	3.98	2
Zn(II)HCF(II)	2:1	1.59:0.82:1		50.62	27.33	22.06	99
PEI–Zn(III)HCF(II)	2:1.5	2:1	120	68.78	31.22	-	44
PEI–Zn(III)HCF(II) monolith [24]	1:1	1.86:0.33:1	114	58.21	31.35	10.44	84
Cu(II)HCF(III)	3:2	2.95:0.09:2		62.43	35.89	1.68	88
PEI–Cu(II)HCF(III)	1:1	2.2:1		69.19	30.81	-	3
Zn(II)HCF(III)	3:2	2.85:0.3:2		55.34	38.83	5.83	99
PEI–Zn(II)HCF(III)	1:1	3:2	120	60.72	39.28	-	40
Fe(III)HCF(III)	1:1			-	99.85	0.15	84
PEI–Fe(III)HCF(III)			33	-	99.75	0.25	6

^1^ Content of Cu(II), Zn(II) or Fe(III) in PEI after the first stage of hybrids synthesis, ^2^ Efficiency of Cs^+^ uptake from solution containing 20 mgCs/L (pH~6) at solid: liquid ratio 1:1000.

**Table 2:** Mossbauer parameters for crystalline HCFs and PEI–HCF hybrids, T = 298 K *.

Sample	δ, mm/s	Δ, mm/s	Γ, mm/s	Relative Area, %	Assignment
Cu(II)HCF(II)	−0.10	-	0.36	100	LS Fe^II^–CN–Cu^II^
PEI–Cu(II)HCF(II)	−0.09	-	0.38	100	LS Fe^II^–CN–Cu^II^–PEI
Zn(II)HCF(II)	−0.12	-	0.26	100	LS Fe^II^–CN–Zn^II^
PEI–Zn(II)HCF(II)	−0.09	-	0.36	100	LS Fe^II^–CN–Zn^II^–PEI
K_4_[Fe(CN)_6_]	−0.05	-	0.31	100	LS Fe^II^–CN–K^I^
Cu(II)HCF(III)	−0.07	-	0.38	7	LS Fe^II^–CN–Cu^II^
	−0.16	0.55	0.35	93	LS Fe^III^–CN–Cu^II^
PEI–Cu(II)HCF(III)	−0.08	-	0.36	100	LS Fe^II^–CN–Cu^II^–PEI
Zn(II)HCF(III)	−0.17	-	0.39	100	LS Fe^III^–CN–Zn^II^
PEI–Zn(II)HCF(III)	−0.09	-	0.38	100	LS Fe^II^–CN–Zn^II^–PEI
Fe(III)HCF(III)	−0.15	-	0.59	54	LS Fe^III^–CN
	0.39	0.34	0.39	46	HS Fe^III^–NC
PEI–Fe(III)HCF(III)	−0.07	-	0.37	35	LS Fe^II^–CN
	0.36	0.53	0.33	37	PEI–HS Fe^III^–NC
	0.35	0.83	0.44	28	HS Fe(III)
K_3_[Fe(CN)_6_]	−0.13	0.28	0.27	100	LS Fe^III^–CN–K^I^
PEI–Fe(III) complex	0.35	0.59	0.34	53	HS Fe(III)
	0.35	0.97	0.39	47	HS Fe(III)

* Isomer shift (δ), quadrupole splitting (Δ), linewidth (Γ). Values of δ are reported relative to α-Fe metal. Fitting error in the values of δ, Δ and Γ remained below 0.01 mm/s.

## Data Availability

Row experimental data are available from the authors upon request.

## Supplementary Materials

The following supporting information can be downloaded at: https://www.mdpi.com/article/10.3390/molecules27238489/s1, Figure S1: XRD patterns of crystalline hexacyanoferrates (HCF) (a) and related PEI-HCF hybrids (b) obtained using K_3_[Fe^III^(CN)_6_] as precursors; Figure S2: Temperature dependence of the magnetization for (a) crystalline HCF(III) and (b) PEI-HCF(III) hybrids; Figure S3: The magnetization curve for PEI-Cu(II)HCF(III) hybrid measured at T = 300 K
