# Supplementary material for: Polyethylenimine as a Non-Innocent Ligand for Hexacyanoferrates Immobilization
Source: Molecules. 2022 Dec 2;27(23):8489. doi: 10.3390/molecules27238489 (PMC9740449; doi:10.3390/molecules27238489)
Supplement: Supplementary file 1 [file molecules-27-08489-s001.zip › molecules-2032433-supplementary.pdf]

Supplementary materials

Polyethylenimine as a non-innocent ligand for hexacyanoferrates immobilization

Denis Balatskiy, Ivan Tkachenko, Irina Malakhova, Natalia Polyakova, and Svetlana Bratskaya*

[1] Institute of Chemistry, Far Eastern Branch of Russian Academy of Sciences, 159, prosp00-letiya Vladivostoka, 690022 Vladivostok, Russia

* Correspondence: sbratska@ich.dvo.ru

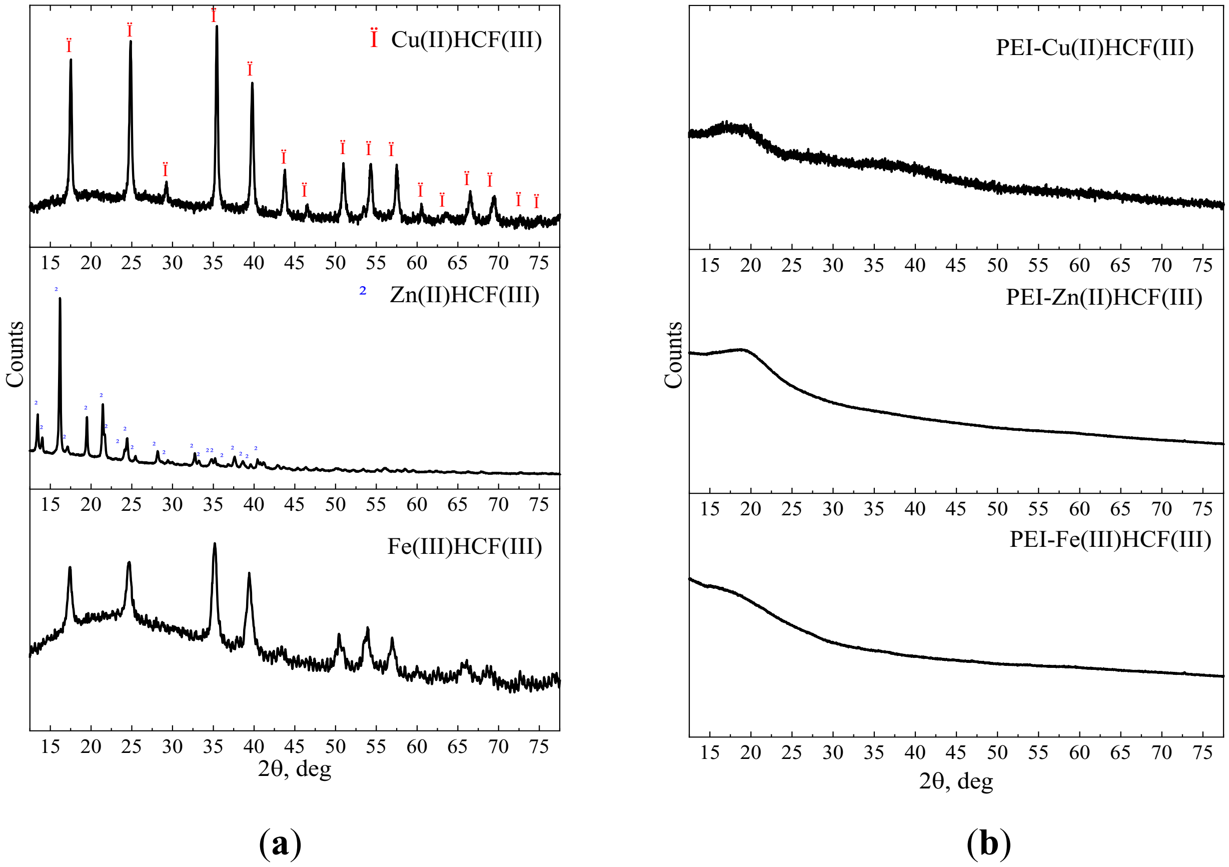

Figure S1. XRD patterns of crystalline hexacyanoferrates (HCF) (a) and related PEI-HCF hybrids (b) obtained using $K_3[Fe^{III}(CN)_6]$ as precursors

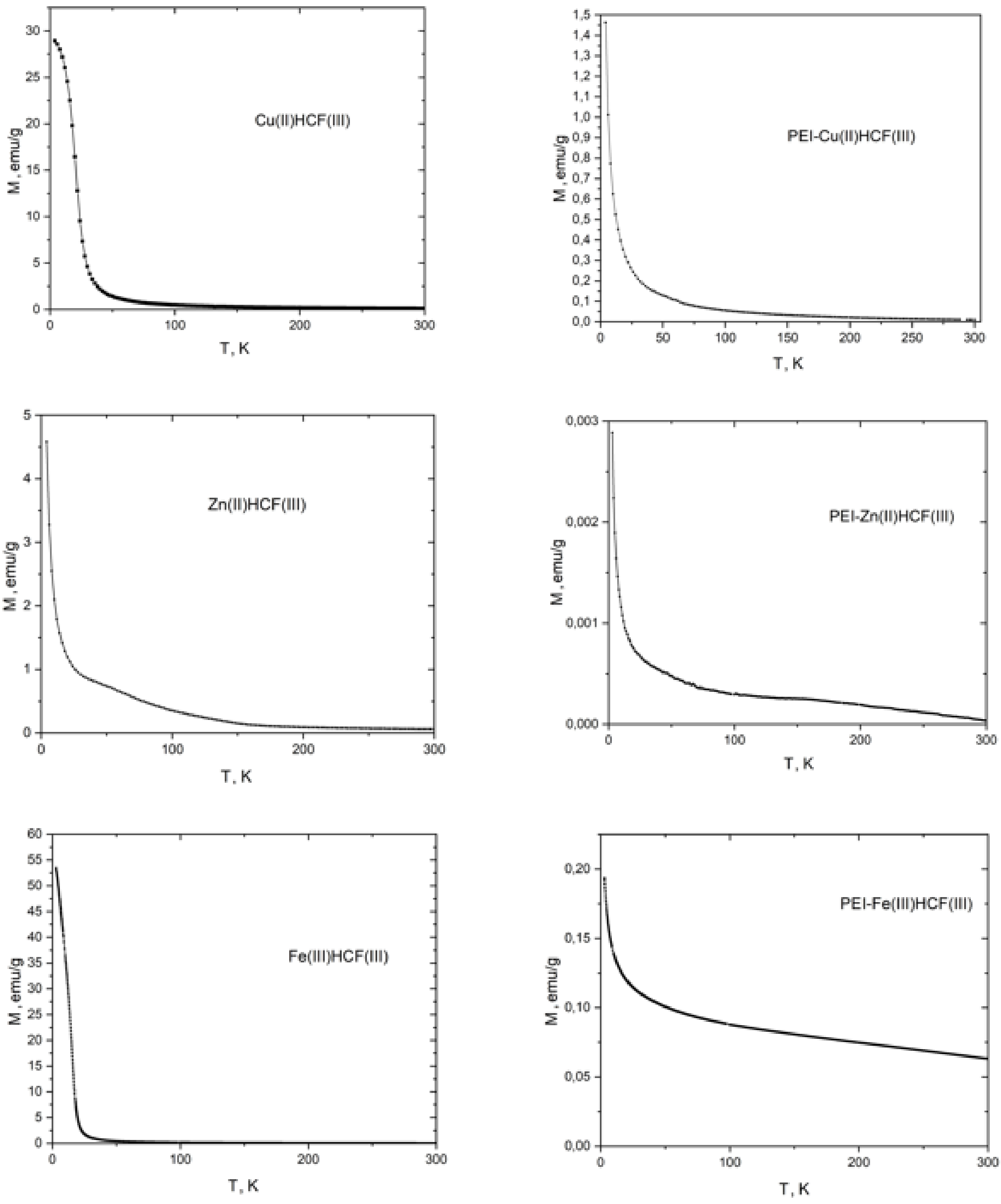

(a) **(b)**

Figure S2. Temperature dependence of the magnetization for (a) crystalline HCF(III) and (b) PEI-HCF(III) hybrids

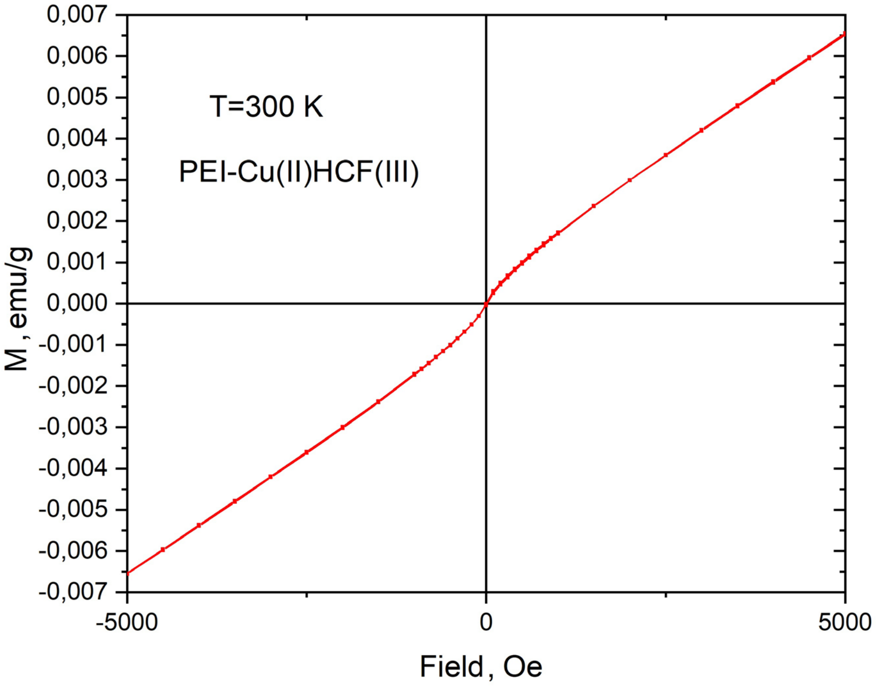

Figure S3. The magnetization curve for PEI-Cu(II)HCF(III) hybrid measured at T=300 K
